# Editorial: *In vivo* applications of nanozymes

**DOI:** 10.3389/fbioe.2026.1852320

**Published:** 2026-04-23

**Authors:** Bing Jiang

**Affiliations:** 1 Nanozyme Laboratory in Zhongyuan, School of Basic Medical Sciences, Zhengzhou University, Zhengzhou, China; 2 Nanozyme Laboratory in Zhongyuan, Henan Academy of Innovations in Medical Science, Zhengzhou, Henan, China

**Keywords:** biomedical applications, in vivo catalysis, microenvironment-responsive, nanozymes, reactive oxygen species regulation

Nanozymes are increasingly moving from proof-of-concept catalytic systems to disease-oriented therapeutic platforms. The defining Research Topic for *in vivo* translation is no longer whether nanomaterials can mimic enzymes under simplified conditions, but whether they can function reliably within heterogeneous pathological microenvironments. The six contributions collected in this Research Topic show that progress clearly. Across four original investigations and two reviews, the Research Topic reveals a field shaped by three converging priorities: microenvironment-specific activation, multifunctional therapeutic integration, and translationally relevant evaluation.

Several contributions demonstrate how nanozymes can serve as regulators of oxidative stress and inflammation in non-malignant disease. Li et al. developed a chitosan–Prussian blue nanozyme for noise-induced hearing loss that reduced intracellular ROS, oxidative damage, apoptosis, and TLR4/NF-κB-mediated inflammatory signaling in cochlear cells, while also mitigating inflammatory activation *in vivo* after round-window delivery. In renal ischemia–reperfusion injury, Liu et al. reported ultrasmall ruthenium/curcumin coordination polymer nanodots with broad radical-scavenging capacity, efficient renal distribution, and protective effects against both acute injury and progression to chronic kidney disease. By reducing oxidative stress, inflammatory infiltration, and fibrosis-associated markers, this work extends the therapeutic scope of nanozymes from acute rescue to longer-term disease modulation. These studies underscore an important point: *in vivo* nanozymes are not only pro-oxidant catalytic tools, but can also function as antioxidant and anti-inflammatory therapeutic modulators when matched to the underlying pathology.

A second set of studies highlights the opposite, but equally important, therapeutic strategy: selectively amplifying oxidative damage at diseased sites. Liang et al. described PdRu@PEI bimetallic nanoalloys that integrate peroxidase-like activity, glutathione depletion, and near-infrared photothermal conversion, thereby enhancing hydroxyl radical generation and antitumor efficacy *in vivo*. Yuan et al. introduced an ultrafast, room-temperature route to L-His-Fe_3_O_4_ nanozymes with enhanced peroxidase-like activity, improved antibacterial performance against both Gram-positive and Gram-negative bacteria, effective biofilm disruption, and accelerated healing of MRSA-infected wounds. In both cases, catalytic activity alone was not sufficient; therapeutic efficacy emerged from rational coupling of enzyme-like reactions with additional design features, including electron-transfer engineering, amino-acid modification, photothermal enhancement, and membrane-disruptive antibacterial action. This progression from single-function catalysis to integrated therapeutic design is one of the clearest themes of the Research Topic.

The two review articles expand this experimental landscape by addressing the design logic needed for broader clinical translation. Fu et al. frame diabetic foot ulcer therapy as a multicomponent challenge involving oxidative stress, infection, chronic inflammation, impaired angiogenesis, and extracellular matrix dysregulation, and argue that coated cerium oxide nanozymes may address these interacting barriers more effectively than unmodified materials. In parallel, Zhuang et al. review pH-responsive nanozymes as smart catalytic systems whose activity can be switched by pathological acidity, showing how surface modification, doping, and core–shell engineering may improve specificity across cancer, infection, wound healing, and inflammatory disease. Taken together, these reviews make clear that future progress will depend not only on discovering new catalytic nanomaterials, but also on improving how nanozymes are stabilized, targeted, activated, and evaluated *in vivo*.

Viewed as a whole, the Research Topic suggests that “*in vivo* applications” should not be understood simply as moving an established nanozyme from the bench into an animal model. Rather, it demands that catalytic behavior be redefined around biological context. First, enzyme-mimetic activity must be matched to real disease microenvironments, including acidic tumors, ROS-rich ischemic tissues, inflamed cochleae, infected wounds, and metabolically compromised chronic ulcers. Second, therapeutic benefit increasingly depends on multifunctionality, with catalytic activity being integrated with drug delivery, photothermal conversion, immunomodulation, oxygen regulation, or anti-fibrotic action. Third, translational relevance depends on practical variables such as delivery route, organ distribution, biosafety, coating strategy, synthesis simplicity, and long-term tolerance.

At the same time, the Research Topic also highlights unresolved challenges. Nanozyme activity *in vivo* remains highly dependent on substrate availability, pathological heterogeneity, and dynamic physicochemical changes that are difficult to model in simplified systems. Standardized frameworks for pharmacokinetics, biodegradation, chronic toxicity, and interstudy comparability remain insufficient. More clinically aligned disease models, deeper mechanistic analysis, and manufacturing-conscious design will therefore be essential if promising preclinical findings are to move beyond proof-of-concept.

**FIGURE 1:**
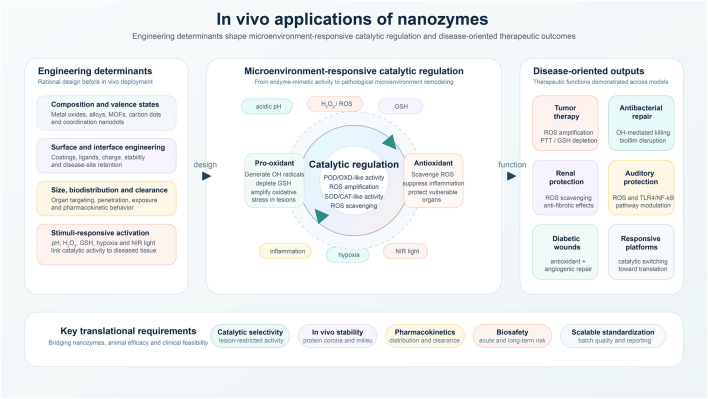
Schematic overview of in vivo applications of nanozymes. Rational engineering of nanozyme composition, surface/interface properties, biodistribution, and stimuli-responsive activation enables microenvironment-responsive catalytic regulation *in vivo*. Depending on pathological context, nanozymes may amplify ROS for tumor therapy and antibacterial treatment, scavenge ROS for organ protection and inflammation control, or integrate catalytic switching with regenerative functions. Translation of nanozyme-based medicine requires improved catalytic selectivity, *in vivo* stability, pharmacokinetics, biosafety, and scalable standardization.

Overall, the contributions gathered in this Research Topic capture a field in transition—from showing that nanozymes can work *in vivo* to defining how they can work selectively, safely, and reproducibly in complex disease settings. They collectively suggest that the future of nanozyme medicine lies in rationally engineered, microenvironment-responsive systems that do more than catalyze reactions: they regulate pathological microenvironments and integrate multiple therapeutic functions (Figure 1). We hope this Research Topic will stimulate further efforts to connect catalytic mechanism with biological complexity and accelerate the translation of nanozymes toward clinically meaningful interventions.

